# Pictorial Review of Rare Pancreatic Tumors and Tumor-like Lesions: Radiologic–Pathologic Correlation

**DOI:** 10.3390/medicina60111766

**Published:** 2024-10-28

**Authors:** Jun Hyung Hong, Jin Woong Kim, Eun Ju Yoon, Sang Gook Song, Hyun Chul Kim, Young Hoe Hur, Hyung Joong Kim

**Affiliations:** 1 Department of Radiology, Chosun University Hospital, Chosun University College of Medicine, Gwangju 61453, Republic of Korea; radhong@chosun.ac.kr (J.H.H.); jw4249@gmail.com (J.W.K.);; 2 Department of Hepato-Biliary-Pancreas Surgery, Chonnam National University Hwasun Hospital, Chonnam National University Medical School, Gwangju 61469, Republic of Korea; 3 Medical Science Research Institute, Kyung Hee University Hospital, Seoul 02447, Republic of Korea

**Keywords:** pancreas, neoplasms, non-neoplastic lesion, differential diagnosis, radiologic–pathologic correlation

## Abstract

Rare pancreatic tumors and non-neoplastic tumor-like lesions present a diagnostic challenge due to their uncommon occurrence and overlapping imaging characteristics with more prevalent pancreatic neoplasms. Advances in imaging technologies and diagnostic criteria have contributed to increased detection of these rare entities in clinical practice. This pictorial review focuses on the radiologic–pathologic correlation of rare pancreatic tumors, including colloid carcinoma, acinar cell carcinoma, pancreatoblastoma, primary pancreatic lymphoma, and non-neoplastic tumor-like lesions such as hamartomas and inflammatory pseudotumors. Detailed imaging features, such as signal intensities on MRI and enhancement patterns on CT, are correlated with pathological findings to assist in the differential diagnosis. Familiarity with these characteristics is crucial for radiologists to ensure accurate diagnosis and guide appropriate treatment strategies, as management and prognosis significantly differ from common pancreatic neoplasms.

## 1. Introduction

Pancreatic ductal adenocarcinoma (PDAC) accounts for the majority of pancreatic neoplasms. Other more common types include pancreatic endocrine tumors and cystic neoplasms, which are frequently encountered in clinical practice. However, rare tumors and non-neoplastic tumor-like lesions of the pancreas, though less common, are becoming more frequently identified due to improvements in imaging techniques and refinements in diagnostic criteria [1,2,3] (Table 1). These advancements allow for earlier and more accurate detection of rare pancreatic entities, but the lack of comprehensive information and unfamiliarity among clinicians can make diagnosis and management challenging.

The significance of differentiating rare pancreatic tumors from common ones lies in the differing treatment protocols and prognoses associated with each type. Unlike PDAC, which typically carries a poor prognosis, some rare pancreatic tumors, such as colloid carcinoma, have a relatively favorable outlook. Therefore, recognizing the unique imaging features of these rare tumors is critical in avoiding misdiagnosis and providing optimal patient care.

This review aims to provide a comprehensive pictorial summary of rare pancreatic tumors and non-neoplastic tumor-like lesions, correlating imaging findings with pathological characteristics. By familiarizing clinicians with these entities, we hope to contribute to more accurate diagnoses and improved patient outcomes in cases of rare pancreatic neoplasms.

## 2. Colloid Carcinoma

Colloid carcinoma is an uncommon variant of ductal adenocarcinoma, constituting 1–3% of invasive pancreatic adenocarcinomas [4]. This neoplasm produces large volumes of mucin, resulting in a cystic appearance in imaging studies [5]. Pathologically, colloid carcinomas are characterized by abundant stromal mucin lakes with scant malignant cells floating within. The prognosis of colloid carcinoma is better than that of ordinary ductal adenocarcinoma.

### 2.1. Imaging Finding

**Figure 1:**
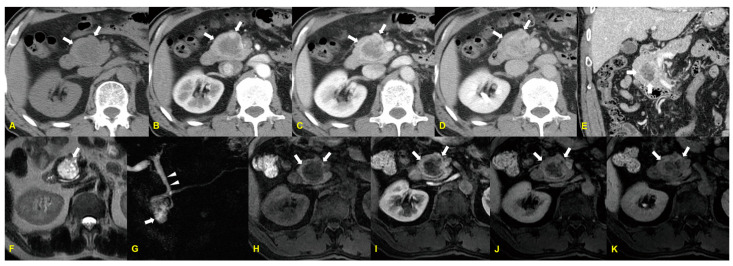
A 65-year-old man with colloid carcinoma. The precontrast axial CT image (**A**) shows a low attenuated mass in the pancreatic head (arrows). Contrast-enhanced arterial (**B**), portal (**C**), delayed (**D**) phase, and coronal reformation (**E**) CT images demonstrate peripheral enhancement on arterial phase images and internal mesh-like enhancement on portal and delayed phase images (arrows in (**B**–**E**)). T2-weighted MR (**F**) and 3D MR cholangiopancreatography (**G**) images demonstrate typical salt-and-pepper appearance (arrow in (**F**,**G**)) and the absence of communication of the mass with the pancreatic duct. The mass caused mild dilatation of the common bile duct (arrowheads in (**G**)). The unenhanced T1-weighted MR image (**H**) and gadolinium-enhanced arterial (**I**), portal (**J**), and delayed (**K**) phase T1-weighted MR images show a progressively peripheral and internal sponge-like enhancement pattern (arrows).

Colloid carcinoma appears as ill-defined or well-defined cystic or non-cystic masses with or without calcification and pancreatic ductal dilatation on CT [5]. It is thought that well-circumscribed lakes of mucin containing neoplastic cells limit further infiltration into the adjacent stroma [6]. MRI demonstrates very high signal intensity or a “salt and pepper” appearance on T2-weighted MR images, along with variable signal intensities on T1-weighted MR images. Small nodular lesions with high signal intensity on T2-weighted MR images correlate with mucin pools. Mesh-like internal low-signal-intensity and tiny hypointense foci on T2-weighted MR images reflect fine stroma and neoplastic cells, respectively, producing the “salt and pepper” appearance. Dynamic imaging shows progressive delayed peripheral and internal sponge-like or mesh-like enhancement of the intervening stroma, with poor enhancement of the mucin pools [5] (Figure 1).

### 2.2. Differential Diagnosis

On T2-weighted MR images, colloid carcinoma exhibits strong high signal intensity due to its abundant extracellular mucin content. Additionally, this tumor does not communicate with the main pancreatic duct, although the duct may be dilated. These imaging features can help differentiate colloid carcinoma from other pancreatic tumors, such as PDAC or intraductal papillary mucinous neoplasms (IPMNs).

## 3. Acinar Cell Carcinoma

Although acinar cells constitute the majority of the pancreas, acinar cell carcinoma (ACC; The World Health Organization 11th Revision of the International Classification of Diseases (ICD-11) code 2C10.0 & XH3PG9) accounts for approximately 1–2% of all pancreatic tumors [7]. ACC typically occurs between the fifth and seventh decades of life [8]. It is characterized by the systemic release of pancreatic enzymes, including trypsin, chymotrypsin, amylase, and lipase. ACC is twice as common in men as it is in women. While the prognosis of ACC is better than that of PDAC, it is worse than that of neuroendocrine tumors [8].

### 3.1. Imaging Finding

**Figure 2:**
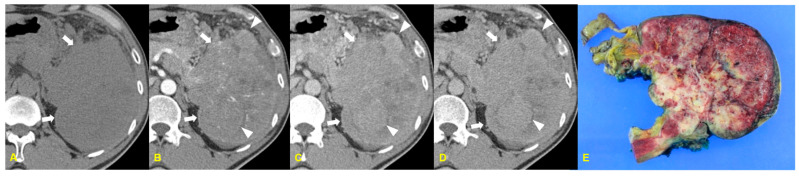
A 54-year-old man with acinar cell carcinoma. The unenhanced CT image (**A**) demonstrates an exophytic mass in the pancreatic tail (arrows). The contrast-enhanced arterial (**B**), portal (**C**), and delayed (**D**) phase CT images demonstrate a heterogeneously enhancing mass (arrows), which has hypodense areas and nodular components (arrowheads). The photograph of the resected specimen (**E**) shows a well-circumscribed reddish mass with a nodular appearance.

ACC tends to be solitary and is more commonly found in the head of the pancreas. These tumors are often exophytic, hypovascular, and well marginated on CT and MRI [8]. Chiou et al. [9] described the typical appearance of ACC as a solitary, well-defined, heterogeneously hypodense mass with a well-defined enhancing capsule in 60% of cases on arterial phase images. Central hypodensity is frequently observed, and internal calcification is also commonly associated with this tumor. According to Tatli et al. [10], over 90% of cases showed well-marginated and exophytic contours (Figure 2).

### 3.2. Differential Diagnosis

The imaging features of ACC are variable, requiring differentiation from other tumors such as PDAC, neuroendocrine tumors, and solid pseudopapillary tumors (SPTs). PDAC is usually smaller, lacks calcifications and central hypodensity, and tends to be locally invasive. Neuroendocrine tumors need to be distinguished from ACC because they typically exhibit a central hypodense area on imaging. However, neuroendocrine tumors are generally more hypervascular than ACC. In some cases, nonfunctioning neuroendocrine tumors may not be distinguishable from ACC. SPTs can also mimic ACC. Both tumors often present as well-marginated, encapsulated masses with both solid and cystic components. However, SPTs occur almost exclusively in young women and tend to have a more heterogeneous and complex appearance.

## 4. Pancreatoblastoma

Pancreatoblastoma (ICD-11 code 2C10.0 & XH27L5) is a rare primary malignant neoplasm of the pancreas that typically affects children between the ages of 1 and 8 years, although cases have also been reported in neonates and adults [7]. The tumor is slightly more common in males, with half of all cases occurring in Asians [11]. Pancreatoblastoma is a slow-growing tumor that can arise from any part of the pancreas [12]. In the absence of distant metastasis, the prognosis is significantly better than that of PDAC.

### 4.1. Imaging Finding

**Figure 3:**
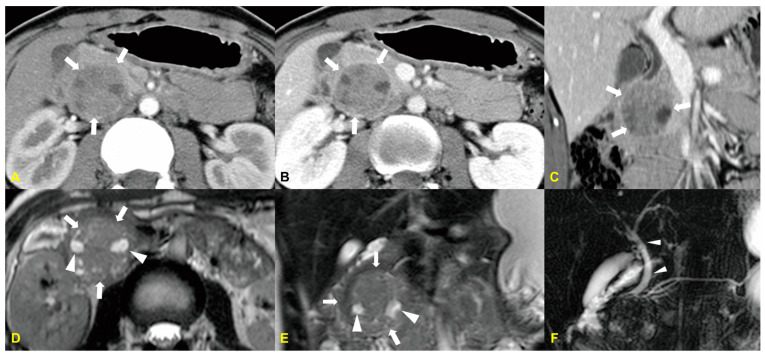
An 18-year-old woman with pancreatoblastoma. The contrast-enhanced arterial (**A**), delayed (**B**) phase, and coronal (**C**) CT images reveal a well-defined mass (arrows) with cystic and enhancing solid components in the head of the pancreas. The T2-weighted MR axial (**D**) and coronal (**E**) MR images show a heterogeneous mass (arrows) with internal high-signal-intensity foci (arrowheads), indicating cystic components. The 3D MR cholangiopancreatography image (**F**) reveals that the mass caused mild dilatation of the extrahepatic bile duct (arrowheads).

Radiologic features typically include well-defined, lobulated, heterogeneous masses with necrosis and/or calcification [13]. On MRI, the tumor shows low-to-intermediate signal intensity on T1-weighted MR images and high signal intensity on T2-weighted MR images. Pancreatoblastomas usually arise from the body and/or tail of the pancreas or involve the entire organ, rather than being located in the pancreatic head [13]. The mass is often large at initial presentation, which can make it difficult to determine the organ of origin. In the series by Montemarano et al. [14], the imaging appearance suggested the pancreas as the origin in only half of the cases. These large tumors typically compress surrounding structures without notable signs of invasion on imaging, although local invasion may be evident during surgical resection [14] (Figure 3).

### 4.2. Differential Diagnosis

The differential diagnosis includes pancreatic abscess, pseudocyst, and other pancreatic neoplasms. Abscesses and pseudocysts are typically characterized by their predominantly cystic appearance. Pancreatic tumors are extremely rare in children; therefore, pancreatoblastoma should be considered in the differential diagnosis of pediatric abdominal masses, especially when the pancreas is suspected as the organ of origin. In adults, various pancreatic tumors such as PDAC, SPTs, and neuroendocrine tumors should be included in the differential diagnosis.

## 5. Primary Leiomyosarcoma

Leiomyosarcoma of the pancreas is a rare malignant tumor and its incidence is only 0.1% of all malignant pancreatic tumors [15]. Leiomyosarcomas originating from the stomach, duodenum, and retroperitoneal organs often invade the pancreas, simulating a primary pancreatic tumor [15]. The diagnosis of pancreatic leiomyosarcoma is confirmed by ruling out tumors arising from surrounding organs. Pancreatic leiomyosarcoma is thought to originate either from the smooth muscle elements of the pancreatic ducts or the walls of small intrapancreatic vessels.

### 5.1. Imaging Finding

**Figure 4:**
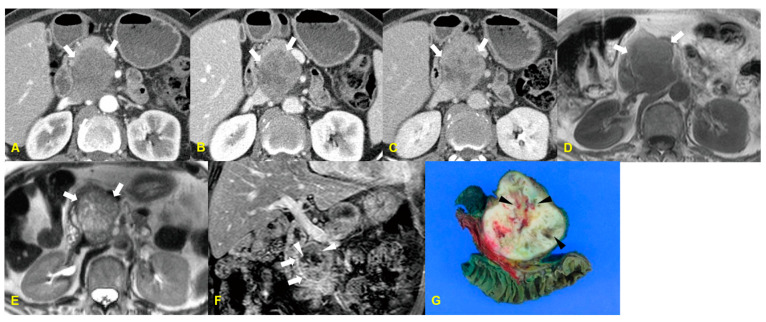
A 74-year-old woman with pancreatic leiomyosarcoma. The contrast-enhanced arterial (**A**), portal (**B**), and delayed (**C**) phase CT images show a lobulated well-defined mass with heterogeneous and delayed enhancement (arrows). MR images demonstrate a well-defined mass with low signal intensity on the T1-weighted MR image ((**D**), arrows) and heterogeneously high signal intensity on the T2-weighted MR image ((**E**), arrows). The mass shows a heterogeneous enhancement pattern with internal cystic portions (arrowheads) and a peripheral enhancing rim (arrows) on the gadolinium-enhanced coronal MR image (**F**). The photograph of the resected specimen (**G**) shows a lobulated and well-defined yellowish mass with internal cystic areas (black arrowheads).

The tumor tends to be large at the time of diagnosis, often showing cystic changes and, in some cases, widespread metastasis without involving the lymph nodes [15]. When pancreatic leiomyosarcoma is small, ultrasonography (US) usually shows a hypoechoic pattern. As the tumor size increases, necrotic or hemorrhagic changes occur, leading to a mosaic pattern on imaging. Paciorek et al. [16] reported that MRI characteristics of pancreatic leiomyosarcomas are best visualized on T1-weighted MR images, where the tumor shows diffuse homogeneous low signal intensity. The mass is less conspicuous on T2-weighted MR images but still appears as a hyperintense lesion in the mid-body of the pancreas. Pancreatic leiomyosarcoma is also described as a hypervascular tumor [15]. In some cases, peripheral enhancement of the tumor may be observed (Figure 4).

### 5.2. Differential Diagnosis

Several imaging features, such as an encapsulated margin, expansive growth, and rare lymphatic involvement, can aid in the differential diagnosis and favor the diagnosis of primary pancreatic leiomyosarcoma.

## 6. Primary Pancreatic Lymphoma (PPL)

Primary pancreatic lymphoma (PPL) is extremely rare, representing less than 0.5% of all pancreatic tumors. The majority of PPLs are intermediate- to high-grade diffuse large-cell lymphomas. The disease occurs more frequently in men and usually presents between the fifth and sixth decades of life. PPL originates within the pancreas, with or without the involvement of the peripancreatic lymph nodes [17]. The diagnostic criteria for PPL include the absence of palpable superficial lymphadenopathy, the absence of mediastinal lymphadenopathy on chest radiography, a normal leukocyte count in peripheral blood, a primary mass in the pancreas with lymph nodal involvement confined to the peripancreatic region, and no hepatic or splenic involvement [18].

### 6.1. Imaging Finding

**Figure 5:**
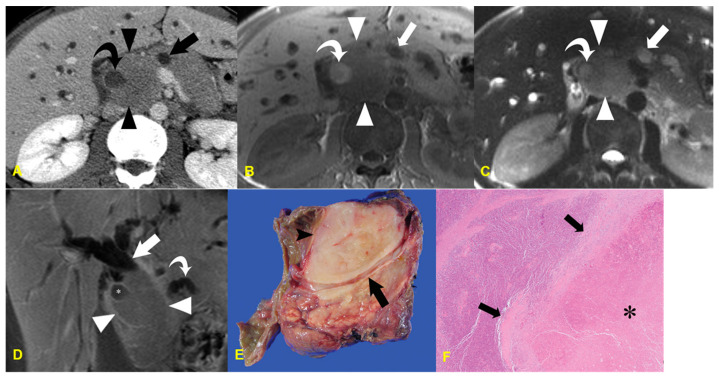
A 27-year-old man with primary pancreatic lymphoma. The contrast-enhanced CT image (**A**) shows a poorly enhancing mass (arrowheads) with an internal cystic portion (curved arrow) in the pancreatic head. Moderate dilatation of the pancreatic duct (arrow) and intrahepatic bile ducts is also noted. MR imaging shows that the mass has a homogeneous low signal intensity and an intermediate signal intensity on T1- and T2-weighted MR images (arrowheads in (**B**,**C**)), respectively. The mass contains a portion of hemorrhagic necrosis (curved arrow in (**B**,**C**)). And also, dilatation of the main pancreatic duct (arrow in (**B**,**C**)) is noted. The gadolinium-enhanced T1-weighted coronal MR image (**D**) shows a well-defined mass (arrowheads) with homogeneous and subtle enhancement, in which hemorrhagic necrosis (asterisk) is included. Moderate dilatation of the main pancreatic duct (curved arrow) and common bile duct (arrow) is noted. The photograph of the resected specimen (**E**) shows a well-circumscribed whitish mass at the pancreatic head, with invasion of the pancreatic duct (arrow) and duodenal wall (arrowhead). The photomicrograph (**F**) shows focal hemorrhagic necrosis (asterisk) with a well-circumscribed margin (arrows) (H & E, × 20).

On CT, two different morphologic patterns of pancreatic involvement can be observed in patients with PPL. The first is a large, localized, well-circumscribed tumoral form with homogeneous enhancement, typically in the pancreatic head, though less commonly a mass may appear in the body or tail of the pancreas. Rarely, a diffusely enlarged, infiltrating, or replacing form occupies most of the pancreatic gland. MR imaging of the well-circumscribed tumoral type demonstrates a homogeneous hypointense mass within the pancreas on T1-weighted MR images with subtle enhancement and a heterogeneous mass with low-to-intermediate signal intensity on T2-weighted MR images. In the case of the diffuse infiltrating type, T1- and T2-weighted MR images show a hypointense, enlarged pancreas with mild-to-moderate enhancement [19] (Figure 5).

### 6.2. Differential Diagnosis

PPL should be differentiated from PDAC and neuroendocrine tumors. PPL typically presents as a well-defined, poorly enhancing mass. Pancreatic duct dilatation tends to be milder compared to PDAC. Neuroendocrine tumors may exhibit necrosis, fibrosis, or cystic degeneration, with hypervascular solid components. Small functioning neuroendocrine tumors usually show early, strong enhancement and are often associated with frequent hepatic metastasis. Low-to-intermediate signal intensity on T2-weighted MR images may be a key imaging feature favoring the diagnosis of PPL.

## 7. Hamartoma

A pancreatic hamartoma is defined as a lesion with disarranged component cells from various organs. This lesion is composed of three disarranged cellular components in varying proportions: acinar, islet, and ductal cells. To our knowledge, 16 cases of pancreatic hamartoma have been reported in the English literature [20,21]. The most common site of pancreatic hamartoma is the pancreatic head; however, it can occur anywhere in the pancreas [21]. Pancreatic hamartomas are categorized as either solid and cystic lesions or solid masses [20].

### 7.1. Imaging Finding

**Figure 6:**
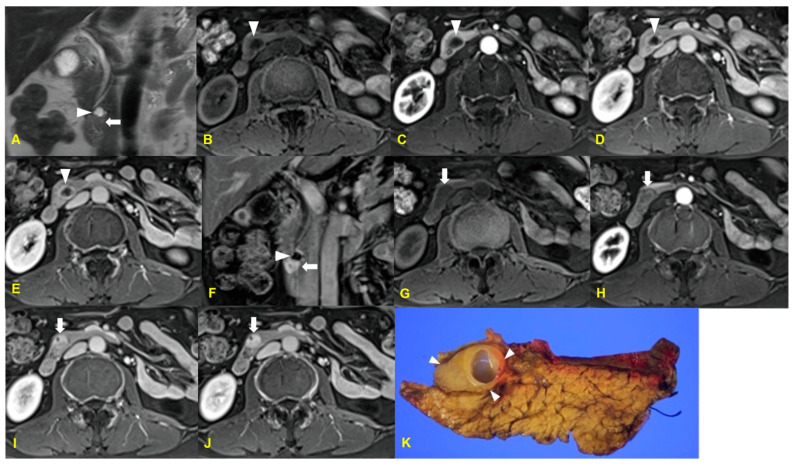
A 52-year-old woman with pancreatic hamartoma. The T2-weighted coronal MR image (**A**) and gadolinium-enhanced T1-weighted coronal MR image (**F**) reveal a mixed solid (arrow) and cystic mass (arrowhead) in the head and uncinate process of the pancreas, with homogeneous enhancement of the solid portion of the mass (arrow in (**F**)). The unenhanced T1-weighted MR images (**B**,**G**) and the gadolinium-enhanced arterial (**C**,**H**), portal (**D**,**I**), and delayed (**E**,**J**) phase T1-weighted MR images demonstrate the cystic portion of the mass with rim enhancement (arrow in (**B**–**E**)) and the solid portion with delayed strong homogeneous enhancement, as compared to the pancreas parenchyma (arrow in (**G**–**J**)). The photograph of the gross surgical specimen (**K**) shows a well-circumscribed yellowish solid and cystic mass in the head and uncinate process of the pancreas (arrowheads).

Imaging findings of pancreatic hamartoma have been described in a few studies. According to previous reports, hamartomas have presented as solid masses in the pancreatic body, well-defined mixed solid and cystic tumors in the pancreatic head, or multicystic masses based on ultrasound and CT imaging [22,23,24]. In contrast, some reports have described hamartomas as ill-defined masses containing cysts and calcifications in the pancreatic head on CT and MRI [21,25]. In our case, the hamartoma appeared as a well-defined mixed solid and cystic mass. The solid portion showed a delayed and strong enhancement pattern, which may be attributable to the presence of a rich fibrous matrix within the lesions (Figure 6).

### 7.2. Differential Diagnosis

It can be difficult to differentiate pancreatic hamartomas from SPTs, cystic neuroendocrine tumors, or cystic neoplasms such as IPMNs. SPTs typically appear as large, well-defined, solid, and cystic masses with areas of hemorrhagic degeneration. However, pancreatic hamartomas are usually smaller in size and lack hemorrhage. While neuroendocrine tumors show early homogeneous enhancement, sometimes with cystic changes when the tumor is large (>8 cm) [26], hamartomas can exhibit cystic changes even when small. IPMNs usually show ductal communication with the mass, which can also be found in pancreatic hamartomas.

## 8. Localized Lymphoid Hyperplasia

Localized lymphoid hyperplasia, also known as “pseudolymphoma,” has been found in various organs, including the skin, orbit, thyroid, breast, lungs, gastrointestinal tract, liver, and pancreas [27]. It is characterized by hyperplastic lymphoid follicles with polymorphic and polyclonal cell populations which are composed of small mature lymphocytes, mature plasma cells, macrophages, and stromal fibrosis. Localized lymphoid hyperplasia of the pancreas is extremely rare. To our knowledge, only four cases of pancreatic localized lymphoid hyperplasia have been reported in the English medical literature [27,28,29,30].

### 8.1. Imaging Finding

**Figure 7:**
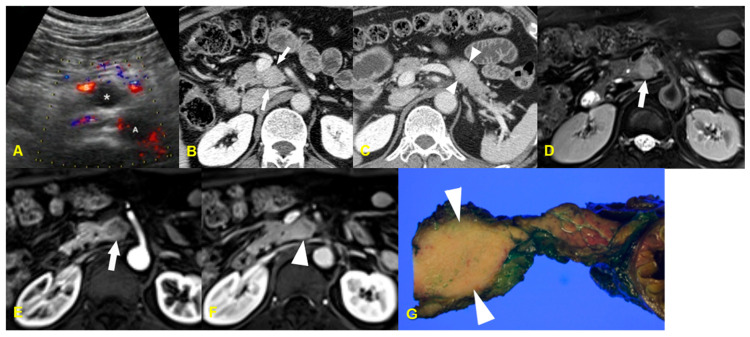
A 52-year-old woman with localized lymphoid hyperplasia of the pancreas. The color Doppler ultrasonographic image (**A**) shows a hypoechoic mass (asterisk) in the uncinate process of the pancreas without discernible vascularity within the mass (A: aorta, S: superior mesenteric vein). The contrast-enhanced delayed phase CT images reveal two localized masses in the uncinate process and tail of the pancreas without peripancreatic stranding (arrows in (**B**) and arrowheads in (**C**)), and these masses show slightly more enhancement, as compared to that of pancreatic parenchyma. The T2-weighted MR image (**D**) shows a localized hyperintense mass without peripancreatic stranding in the uncinate process of the pancreas (arrow). The gadolinium-enhanced dynamic T1-weighted MR images (**E**,**F**) show a slightly hypointense mass (arrow in (**E**)) in the uncinated process of the pancreas on the arterial phase image (**E**), and the mass appears as slightly hyperintense on the portal phase image (**F**). The photograph of resected specimen (**G**) shows a well-circumscribed yellowish mass (arrowheads) in the uncinate process of the pancreas.

The imaging findings of pancreatic localized lymphoid hyperplasia are not well known, making it difficult to differentiate from other pancreatic neoplasms. On ultrasound (US), localized lymphoid hyperplasia has been described as a hypoechoic mass. CT typically shows a well-defined, round mass with delayed enhancement [27,28,29,30]. MRI demonstrates hypointensity on T1-weighted MR images and hyperintensity on T2-weighted MR images. Gadolinium-enhanced MR images reveal arterial hypovascularity with delayed enhancement [28] (Figure 7).

### 8.2. Differential Diagnosis

The differential diagnosis of localized lymphoid hyperplasia includes neuroendocrine tumors, autoimmune pancreatitis, lymphoma, and SPTs. Neuroendocrine tumors can be differentiated from localized lymphoid hyperplasia by their characteristic strong early enhancement. The localized form of autoimmune pancreatitis may present as a focal mass-like lesion, sharing imaging findings, such as delayed enhancement and a nondilated pancreatic duct, with localized lymphoid hyperplasia [31]. However, the localized form of autoimmune pancreatitis is associated with wall thickening and the enhancement of the common bile duct, along with upstream bile ductal dilatation [31]. Although pancreatic lymphoma manifests as a well-defined mass without pancreatic duct dilatation, low-to-intermediate signal intensity on T2-weighted MR images is suggestive of PPL [18].

## 9. Autoimmune Pancreatitis

Autoimmune pancreatitis (ICD-11 code DC33) has been described by several names, including lymphoplasmacytic sclerosing pancreatitis, autoimmune pancreatitis, and sclerosing pancreatitis [7]. In autoimmune pancreatitis, periductal infiltration of IgG4-positive plasma cells leads to periductal and interlobular fibrosis. This fibrosis eventually causes diffuse narrowing of the pancreatic duct and acinar atrophy. According to the diagnostic criteria suggested by Chari et al. [32], a diagnosis of autoimmune pancreatitis is made in patients with one or more of the following findings: (a) histologic findings diagnostic for autoimmune pancreatitis, (b) characteristic CT and pancreatographic findings with elevated serum IgG4 levels, and (c) a response to steroid therapy. Autoimmune pancreatitis accounts for 2–11% of all chronic pancreatitis cases [32]. The disease occurs twice as frequently in men as it does in women, with an age at presentation ranging from 14 to 85 years, and a mean age of over 60 years [33].

### 9.1. Imaging Finding

**Figure 8:**
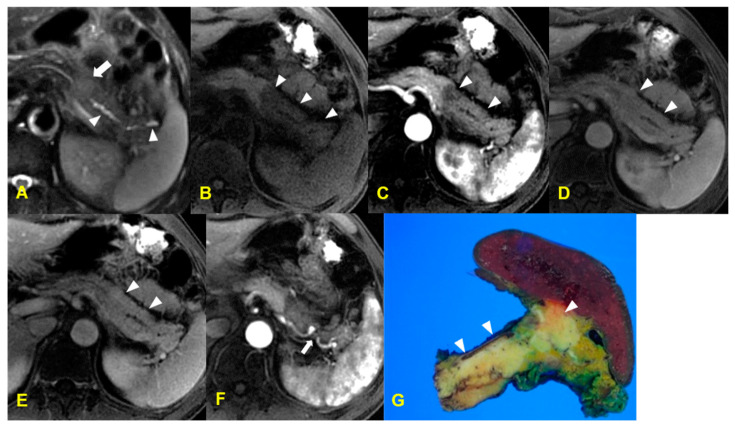
A 58-year-old man with autoimmune pancreatitis. The T2 weighted MR image (**A**) shows an ill-defined focal hyperintense lesion in the pancreatic body (arrow), causing dilatation of the upstream pancreatic duct (arrowheads). The T1-weighted MR image (**B**) shows diffuse low signal intensity covering the body and tail of the pancreas (arrowheads). The gadolinium-enhanced arterial (**C**), portal (**D**), and delayed (**E**) phase T1-weighted MR images demonstrate homogeneous and delayed enhancement (arrowheads) with minimal peripancreatic stranding. The gadolinium-enhanced arterial phase T1-weighted MR image (**F**) shows focal ectasia of the splenic artery (arrow), which suggests possible arterial invasion by the pancreatic lesion. The photograph of the resected specimen (**G**) demonstrates that pancreas parenchyma is replaced by a yellowish mass-like lesion in the body and tail of the pancreas (arrowheads).

There are three different patterns of autoimmune pancreatitis: diffuse, focal, and multifocal. Among these, the diffuse type is the most common, characterized by a diffusely enlarged, sausage-like appearance with sharp margins, a loss of lobular contour, and the absence of pancreatic clefts. Less commonly, the focal type appears as a well-defined, mass-like lesion in the pancreatic head. Multifocal involvement may also occur. Ultrasound and CT reveal hypo-echogenicity and hypo-attenuation of the affected lesions, respectively. MRI demonstrates mild hyperintensity on T2-weighted MR images and hypointensity on T1-weighted MR images. On dynamic enhanced images, poor enhancement during the early arterial phase and moderate enhancement during the delayed phase are typically seen, likely due to fibrosis in the affected area [34]. The presence of a capsule-like rim or “halo” is common in patients with autoimmune pancreatitis and is thought to represent fluid, phlegmon, or fibrous tissue [35]. Lesions in the pancreatic head are typically accompanied by irregular narrowing of the intrapancreatic portion of the common bile duct. Peripancreatic lymphadenopathy is occasionally observed, while pancreatic calcifications, pseudocyst formation, and peripancreatic stranding are rare [36] (Figure 8).

### 9.2. Differential Diagnosis

The differential diagnosis of the focal type of autoimmune pancreatitis includes PDAC. In general, autoimmune pancreatitis is characterized by mild upstream dilatation of the main pancreatic duct. The diffuse type of autoimmune pancreatitis could be mistaken for acute pancreatitis. Minimal or absent peripancreatic stranding is indicative of autoimmune pancreatitis. The relative paucity of peripancreatic fat necrosis and the presence of a peripancreatic halo may also be useful differential diagnostic points from acute pancreatitis.

## 10. Infarcted Intrapancreatic Accessory Spleen

An accessory spleen, a relatively common congenital defect, is observed in 10–30% of patients at autopsy. It most commonly occurs near the splenic hilum, with almost 20% located in or near the pancreatic tail. Accessory spleens can range in size from a few millimeters to several centimeters [37]. They usually appear as isolated, asymptomatic abnormalities but can be associated with other anomalies such as polysplenia or a short pancreas. Torsion and infarction of an intrapancreatic or peripancreatic accessory spleen is extremely rare, with most cases occurring in children. Symptoms can range from vague abdominal discomfort to the acute onset of severe abdominal pain, fever, and vomiting [38].

### 10.1. Imaging Finding

**Figure 9:**
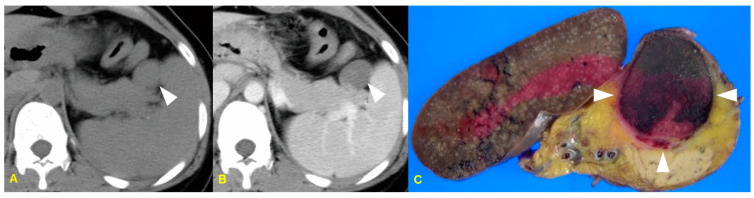
A 32-year-old woman with an infarcted intrapancreatic accessory spleen. The unenhanced CT image (**A**) shows a well-defined oval iso-attenuated mass abutting to the pancreatic tail (arrowhead). The contrast-enhanced CT image (**B**) demonstrates a poorly enhancing mass accompanied by peripheral rim enhancement (arrowhead). The photograph of the resected specimen (**C**) shows a well-defined dark brownish mass (arrowheads).

An intrapancreatic accessory spleen typically manifests as a well-defined round or oval mass. On ultrasound (US), the echogenicity of accessory spleens is similar to that of the spleen. It usually shows posterior acoustic enhancement and a high-amplitude interface due to its fibrotic capsule. On dynamic CT, the enhancement pattern is similar to that of the spleen in all phases, with higher attenuation than the pancreas [39]. The MR signal intensities of intrapancreatic accessory spleens are also similar to those of the spleen. In cases of an infarcted intrapancreatic accessory spleen, precontrast CT can demonstrate an inhomogeneous appearance of the spleen. However, on contrast-enhanced CT, the lesion shows irregular or diffuse low attenuation with poor enhancement. Sometimes, peripheral enhancement of the splenic tissue can be observed due to perfusion via capsular vessels [38] (Figure 9).

### 10.2. Differential Diagnosis

An intrapancreatic accessory spleen can be differentiated from other pancreatic tumors by its enhancement pattern, which is similar to that of the spleen, exhibiting characteristic arciform or zebra-patterned enhancement. When infarction is present, the possibility of an infarcted intrapancreatic accessory spleen should be considered, as indicated by poor enhancement of the solid mass.

## 11. Inflammatory Pseudotumor

An inflammatory pseudotumor, or inflammatory myofibroblastic tumor, is a non-neoplastic tumor-like lesion that is histologically characterized by paucicellular or moderately cellular fibrous tissue with an admixed inflammatory infiltrate of varying density. The ill-defined soft tissue mass may compress or extend into adjacent structures, leading to a variety of symptoms depending on the organs involved. In addition to local pain and symptoms related to the mass lesion, systemic effects such as a loss of appetite, malaise, wasting, and subfebrile temperature elevations may be present. These symptoms occur more frequently in women than in men. The age at presentation ranges from 2.5 to 70 years, with a mean age of 36 years. The lesion size ranges from 1.5 to 13 cm, with an average size of 6 cm. The most common site of pancreatic inflammatory pseudotumors is the head of the pancreas [40].

### 11.1. Imaging Finding

**Figure 10:**
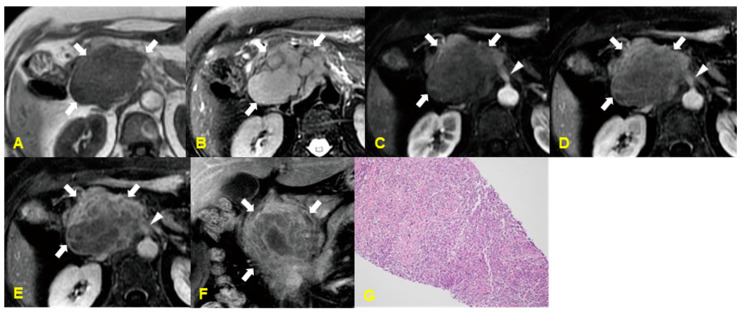
A 52-year-old woman with localized lymphoid hyperplasia of the pancreas. The T1-weighted MR image (**A**) shows a well-defined and lobulated low-signal-intensity mass in the pancreatic head (arrows). On the T2-weighted MR image (**B**), the mass appears as a hyperintense lesion with internal septa (arrows). The gadolinium-enhanced arterial (**C**), portal (**D**), and delayed (**E**) phase and coronal (**F**) T1-weighted MR images demonstrate a heterogeneously enhancing mass (arrows). Somewhat irregular enhancement of the septa is prominently noted on the delayed phase T1-weighted MR image. The mass is interpreted to invade the celiac axis (arrowhead in (**C**–**E**)). The photomicrograph (**G**) shows the proliferation of spindle cells without cytologic atypia, admixed with a chronic inflammatory cell infiltrate (H & E, × 40).

The imaging findings of pancreatic inflammatory pseudotumors are variable. On unenhanced CT, the tumor may appear as a mass with hypo- or iso-attenuation relative to muscle. Calcification can sometimes be observed within the mass. The enhancement pattern is also variable, including delayed peripheral enhancement, heterogeneous, homogeneous, or even no enhancement. Larger lesions may exhibit central necrosis [40,41]. The MRI features of these tumors are similarly variable. The tumor typically appears hypointense on T1-weighted MR images and hyperintense on T2-weighted MR images [41] (Figure 10).

### 11.2. Differential Diagnosis

Due to the variable findings of an inflammatory pseudotumor, it can be difficult to differentiate it from other pancreatic neoplasms. Therefore, inflammatory pseudotumor should be considered a diagnosis only after most other potential tumors have been ruled out.

## 12. Conclusions

The overlapping imaging findings of various pancreatic neoplasms sometimes cause difficulty in differential diagnosis among each disease, especially in rare pancreatic tumors and non-neoplastic tumor-like lesions of the pancreas. Although the imaging features of rare pancreatic tumors and non-neoplastic lesions of the pancreas are not well known, these may be helpful according to previously reported articles (Table 2). It is important for the radiologist to be familiar with the image findings of these disease entities to accomplish an appropriate differential diagnosis, ultimately leading to the optimal treatment strategy for patients. Differentiating rare pancreatic tumors and non-neoplastic lesions from more common neoplasms is essential for determining the appropriate treatment strategy and improving patient outcomes. Despite the limited familiarity with these conditions, several key imaging features have been identified that can aid in the accurate diagnosis of rare pancreatic tumors (Table 2). For instance, colloid carcinoma, with its characteristic cystic appearance due to mucin production, can be distinguished from PDAC and other mucin-producing neoplasms. Similarly, imaging features such as the well-circumscribed nature of acinar cell carcinoma, the large size and lobulated appearance of pancreatoblastoma, and the unique enhancement patterns of primary pancreatic lymphoma can serve as important diagnostic clues. Understanding these imaging–pathologic correlations is crucial for radiologists in making accurate diagnoses.

This review emphasizes the key imaging features of rare pancreatic tumors and non-neoplastic lesions, which are essential for differentiation from common neoplasms. Increasing the awareness of these features helps clinicians guide treatment management and improve patient prognosis. Ongoing advancements in imaging and further research will enhance diagnostic accuracy and care.

## Figures and Tables

**Table 1:** The classification of rare tumors and non-neoplastic tumor-like lesions in the pancreas.

Classification	Incidence
Epithelial tumors	
Colloid carcinoma (mucinous noncystic carcinoma)	1–3% of invasive pancreatic adenocarcinomas
Acinar cell carcinoma	1–2% of all pancreatic neoplasm
Pancreatoblastoma	<200 cases reported
Non-epithelial tumors	
Leiomyosarcoma	0.1% of all pancreatic neoplasm
Lymphomas	0.5% of all pancreatic neoplasm
Non-neoplastic tumor-like lesions	
Hamartoma	16 cases reported in the English literature
Localized lymphoid hyperplasia	4 cases reported in the English literature
Autoimmune pancreatitis	2–11% of all cases of chronic pancreatitis
Intrapancreatic accessory spleen	<20 cases reported
Inflammatory pseudotumor	<30 cases reported

**Table 2:** A summary of the key radiologic features of rare tumors and non-neoplastic tumor-like lesions in the pancreatic region.

	Radiologic Features
Colloid carcinoma	Very high SI and salt-and-pepper appearance on T2WI/ Internal sponge-like or mesh-like enhancement/No communication with pancreatic duct
Acinar cell carcinoma	Solitary/Exophytic and hypovascular mass with well-defined margin/Calcifications (frequent)
Pancreatoblastoma	Well defined and lobulated mass with heterogeneous enhancement/Calcification (±)/ Large tumors without invasion of surrounding structures
Leiomyosarcoma	Well defined and encapsulated mass with heterogeneous enhancement/ Large tumor with invasion of adjacent structure/Lymphadenopathy (rare)
Primary lymphoma	Localized type: well defined mass with poor enhancement/Low-to-intermediate SI on T2WI
	Diffuse infiltrating type: hypointense enlarged pancreas on T1- and T2WI
Hamartoma	Enhancement pattern: variable/sometimes, delayed homogeneous strong enhancement
Localized lymphoid hyperplasia	Well defined mass with delayed enhancement
Autoimmune pancreatitis	Diffuse type: enlarged pancreas with sausage-like appearance
	Focal type: relatively well defined with delayed enhancement
Infarcted intrapancreatic spleen	Enhancement pattern: poor enhancement/ Sometimes, peripheral enhancement by capsular vessels
Inflammatory pseudotumor	Variable image findings

## Data Availability

The data presented in this study are available upon request from the corresponding author. The data are not publicly available for confidentiality reasons.

## References

[1] Bosman F.T., Carneiro F., Hruban R.H., Theise N.D. (2010). WHO Classification of Tumours of the Digestive System.

[2] Hruban R.H., Pitman M.B., Klimstra D.S. (2007). Tumors of the Pancreas (AFIP Atlas of Tumor Pathology. Series 4).

[3] Pauser U., Kosmahl M., Kruslin B., Klimstra D.S., Kloppel G. (2005). Pancreatic solid and cystic hamartoma in adults: Characterization of a new tumorous lesion. Am. J. Surg. Pathol..

[4] Seidel G., Zahurak M., Iacobuzio-Donahue C., Sohn T.A., Adsay N.V., Yeo C.J., Lillemoe K.D., Cameron J.L., Hruban R.H., Wilentz R.E. (2002). Almost all infiltrating colloid carcinomas of the pancreas and periampullary region arise from in situ papillary neoplasms: A study of 39 cases. Am. J. Surg. Pathol..

[5] Yoon M.A., Lee J.M., Kim S.H., Lee J.Y., Han J.K., Choi B.I., Choi J.Y., Park S.H., Lee M.W. (2009). MRI features of pancreatic colloid carcinoma. Am. J. Roentgenol..

[6] Adsay V.N., Merati K., Nassar H., Shia J., Sarkar F., Pierson C.R., Cheng J.D., Visscher D.W., Hruban R.H., Klimstra D.S. (2003). Pathogenesis of colloid (pure mucinous) carcinoma of exocrine organs: Coupling of gel-forming mucin (MUC2) production with altered cell polarity and abnormal cell-stroma interaction may be the key factor in the morphogenesis and indolent behavior of colloid carcinoma in the breast and pancreas. Am. J. Surg. Pathol..

[7] https://icd.who.int.

[8] Hsu M.Y., Pan K.T., Chu S.Y., Hung C.F., Wu R.C., Tseng J.H. (2010). CT and MRI features of acinar cell carcinoma of the pancreas with pathological correlations. Clin. Radiol..

[9] Chiou Y.Y., Chiang J.H., Hwang J.I., Yen C.H., Tsay S.H., Chang C.Y. (2004). Acinar cell carcinoma of the pancreas: Clinical and computed tomography manifestations. J. Comput. Assist. Tomogr..

[10] Tatli S., Mortele K.J., Levy A.D., Glickman J.N., Ros P.R., Banks P.A., Silverman S.G. (2005). CT and MRI features of pure acinar cell carcinoma of the pancreas in adults. Am. J. Roentgenol..

[11] Mergo P.J., Helmberger T.K., Buetow P.C., Helmberger R.C., Ros P.R. (1997). Pancreatic neoplasms: MR imaging and pathologic correlation. Radiographics.

[12] Cavallini A., Falconi M., Bortesi L., Crippa S., Barugola G., Butturini G. (2009). Pancreatoblastoma in adults: A review of the literature. Pancreatology.

[13] Roebuck D.J., Yuen M.K., Wong Y.C., Shing M.K., Lee C.W., Li C.K. (2001). Imaging features of pancreatoblastoma. Pediatr. Radiol..

[14] Montemarano H., Lonergan G.J., Bulas D.I., Selby D.M. (2000). Pancreatoblastoma: Imaging findings in 10 patients and review of the literature. Radiology.

[15] Izumi H., Okada K.I., Imaizumi T., Hirabayashi K., Matsuyama M., Dowaki S., Tobita K., Makuuchi H. (2011). Leiomyosarcoma of the pancreas: Report of a case. Surg. Today.

[16] Paciorek M.L., Ross G.J. (1998). MR imaging of primary pancreatic leiomyosarcoma. Br. J. Radiol..

[17] Lin H., Li S.D., Hu X.G., Li Z.S. (2006). Primary pancreatic lymphoma: Report of six cases. World J. Gastroenterol..

[18] Dawson I.M., Cornes J.S., Morson B.C. (1961). Primary malignant lymphoid tumours of the intestinal tract. Report of 37 cases with a study of factors influencing prognosis. Br. J. Surg..

[19] Merkle E.M., Bender G.N., Brambs H.J. (2000). Imaging findings in pancreatic lymphoma: Differential aspects. Am. J. Roentgenol..

[20] Nagata S., Yamaguchi K., Inoue T., Yamaguchi H., Ito T., Gibo J., Tanaka M., Tsuneyoshi M. (2007). Solid pancreatic hamartoma. Pathol. Int..

[21] Sampelean D., Adam M., Muntean V., Hanescu B., Domsa I. (2009). Pancreatic hamartoma and SAPHO syndrome: A case report. J. Gastrointestin. Liver Dis..

[22] Burt T.B., Condon V.R., Matlak M.E. (1983). Fetal pancreatic hamartoma. Pediatr. Radiol..

[23] Flaherty M.J., Benjamin D.R. (1992). Multicystic pancreatic hamartoma: A distinctive lesion with immunohistochemical and ultrastructural study. Hum. Pathol..

[24] Izbicki J.R., Knoefel W.T., Muller-Hocker J., Mandelkow H.K. (1994). Pancreatic hamartoma: A benign tumor of the pancreas. Am. J. Gastroenterol..

[25] McFaul C.D., Vitone L.J., Campbell F., Azadeh B., Hughes M.L., Garvey C.J., Ghaneh P., Neoptolemos J.P. (2004). Pancreatic hamartoma. Pancreatology.

[26] Buetow P.C., Parrino T.V., Buck J.L., Pantongrag-Brown L., Ros P.R., Dachman A.H., Cruess D.F. (1995). Islet cell tumors of the pancreas: Pathologic-imaging correlation among size, necrosis and cysts, calcification, malignant behavior, and functional status. Am. J. Roentgenol..

[27] Hatzitheoklitos E., Büchler M.W., Friess H., DiSebastiano P., Poch B., Beger H.G., Mohr W. (1994). Pseudolymphoma of the pancreas mimicking cancer. Pancreas..

[28] Kim J.W., Shin S.S., Heo S.H., Jeong Y.Y., Kang H.K., Choi Y.D. (2011). Imaging findings of localized lymphoid hyperplasia of the pancreas: A case report. Korean J. Radiol..

[29] Nakashiro H., Tokunaga O., Watanabe T., Ishibashi K., Kuwaki T. (1991). Localized lymphoid hyperplasia (pseudolymphoma) of the pancreas presenting with obstructive jaundice. Hum. Pathol..

[30] Babaryka I., Thomas E. (1981). Pancreatitis lymphomatosa (author’s transl). Zentralbl. Allg. Pathol..

[31] Kawamoto S., Siegelman S.S., Hruban R.H., Fishman E.K. (2008). Lymphoplasmacytic sclerosing pancreatitis (autoimmune pancreatitis): Evaluation with multidetector CT. Radiographics.

[32] Chari S.T. (2007). Diagnosis of autoimmune pancreatitis using its five cardinal features: Introducing the Mayo Clinic’s HISORt criteria. J. Gastroenterol..

[33] Chari S.T., Smyrk T.C., Levy M.J. (2006). Diagnosis of autoimmune pancreatitis: The Mayo Clinic experience. Clin. Gastroenterol. Hepatol..

[34] Takahashi N., Fletcher J.G., Hough D.M. (2009). Autoimmune pancreatitis: Differentiation from pancreatic carcinoma and normal pancreas on the basis of enhancement characteristics at dual-phase CT. Am. J. Roentgenol..

[35] Vlachou P.A., Khalili K., Jang H.J., Fischer S., Hirschfield G.M., Kim T.K. (2011). IgG4-related sclerosing disease: Autoimmune pancreatitis and extrapancreatic manifestations. Radiographics.

[36] Shanbhogue A.K., Fasih N., Surabhi V.R., Doherty G.P., Shanbhogue D.K., Sethi S.K. (2009). A clinical and radiologic review of uncommon types and causes of pancreatitis. Radiographics.

[37] Sica G.T., Reed M.F. (2000). Case 27: Intrapancreatic accessory spleen. Radiology.

[38] Mendi R., Abramson L.P., Pillai S.B., Rigsby C.K. (2006). Evolution of the CT imaging findings of accessory spleen infarction. Pediatr. Radiol..

[39] Kim S.H., Lee J.M., Han J.K. (2008). Intrapancreatic accessory spleen: Findings on MR Imaging, CT, US and scintigraphy, and the pathologic analysis. Korean J. Radiol..

[40] Pungpapong S., Geiger X.J., Raimondo M. (2004). Inflammatory myofibroblastic tumor presenting as a pancreatic mass: A case report and review of the literature. J. Pancreas.

[41] McClain M.B., Burton E.M., Day D.S. (2000). Pancreatic pseudotumor in an 11-year-old child: Imaging findings. Pediatr. Radiol..

